# Managers’ Conceptions and Their Effects on the Perception of Employees with Disabilities

**DOI:** 10.3390/ijerph17197039

**Published:** 2020-09-25

**Authors:** Antônio Luiz Marques, Marina Romeo, Marjorye Matalinares, Montserrat Yepes-Baldó

**Affiliations:** 1 Economic Sciences Faculty, Centro Universitário Unihorizontes, Belo Horizonte 31270-901, Brazil; marques@face.ufmg.br (A.L.M.); marjoryesom@gmail.com (M.M.); 2 Social Psychology and Quantitative Psychology, Universitat de Barcelona, 08035 Barcelona, Spain; myepes@ub.edu

**Keywords:** diversity management, people with disabilities, labor inclusion, Disability Conceptions Inventory (DCI)

## Abstract

The research aimed to identify managers’ conceptions of disability and the relationship that was established between these conceptions and their perception of the persons with disabilities (PWD) performance, bond, benefits of hiring, and training needs. 257 managers answered a questionnaire in order to identify conceptions of disability in organizations. Descriptive statistics, factorial analysis, and hierarchical analysis of grouping were performed while using IBM Statistic 20.0.0. The results show that managers who have the spiritual and the conception based on inclusion perceive the insertion of PWD as beneficial to the organization. Those who conceive disability as a question of normality perceive the PWD performance as inferior to those without disabilities, which implies that PWDs should be segregated; and, the managers who perceive disability as a social problem are likely to place PWDs in the workplace according to their potential. The results can be fruitfully used by managers, human resources’ professionals, academics, and the society to promote inclusion.

## 1. Introduction

The management of diversity in organizations has been the subject of countless research papers, especially during the last two decades, and centered especially on gender diversity [1,2]. Managers are considered to be the key professionals when it comes to creating an environment that helps each employee to properly fulfill their potential contribution to the organization [3,4]. Effective management and leadership are considered to be critical for raising awareness of the need for inclusion in all employee groups [5].

People with disabilities are the world’s largest minority group. Over a billion people, about 15% of the world’s population, have some form of disability [6,7], and their prevalence is increasing due to the aging population [6,8].

Although there is no universal definition for disability [9], it is described by the International Classification of Functioning (ICF) as a multidimensional phenomenon that arises from a dynamic interaction between people and their physical and social environments [6,10].

The United Nations Convention on the Rights of Persons with Disabilities [11] states, in its Article 1, that: “Persons with disabilities include those who have long-term physical, mental, intellectual or sensory impairments which in interaction with various barriers may hinder their full and effective participation in society on an equal basis with others”.

Despite their growing numbers, persons with disabilities (PWDs) are often subject to discrimination and unfavorable treatment, even in developed countries [12].

The low percentage of formal employment of this social group worldwide [13] has been interpreted for a long time as a consequence of individual disability (medical model of disability). However, recent research based on the social model of disability has shown that the origin of the difficulties and exclusion of this group lie in the way that society perceives and acts when faced by disability [13,14].

More recently, some countries, such as the United Kingdom, have come to realize that negative attitudes toward PWDs integration may result in the loss of a relevant potential resource. Because of these negative attitudes, PWDs may end up affected by a lack of work opportunities, low self-esteem, isolation, and illness, which results in great social burden [15,16,17,18].

While PWDs continue to face significant barriers to the inclusion in the labor market and society, their potential contributions in terms of human capital, business productivity, and innovative capacity are considered to be valuable [19,20]. Fomenting the employment of PWDs is considered as crucial for equality of opportunity, social mobility, and workforce diversity [19].

A systematic review of the literature regarding the benefits of hiring PWDs [20] shows that, as far as the organizations are concerned, the main benefits are improvements in profitability, a competitive advantage, inclusive work culture, and ability awareness. The benefits for the individuals are improved quality of life and income, enhanced self-confidence, an expanded social network, and a sense of community.

In view of these findings, the objective of this research was to identify managers’ conceptions of disability and the relationship that was established between these conceptions and the managers’ perception of PWDs performance, bonding, the benefits of employing PWDs, and training needs.

Following the introduction, this article provides insight into disability literature as a universal phenomenon, its historical and current conceptions, and the impact of prejudice and discrimination on the inclusion of PWDs in the world of work. Secondly, the research procedures adopted for data collection and analysis are described. Thirdly, the results, discussion, and implications of the empirical study are presented. Finally, the conclusions, contributions of the study, and recommendations for future research on the subject are highlighted.

## 2. Conceptual Framework

### 2.1. Disability: Prejudice and Discrimination

As Professor Stephen Hawking stated, “disability needs not be an obstacle to success. I have had motor neuron disease for practically all my adult life. Yet it has not prevented me from having a prominent career in astrophysics and a happy family life” [6] (p. ix). Nonetheless, he recognized that his situation was very different from most people with disabilities, because his success in theoretical physics ensured him adequate support “to live a worthwhile life” [6] (p. ix).

Disability is part of the human condition. Almost all humans will suffer disability either temporarily or permanently at some point in their lives. More than one-billion people worldwide live with some sort of disability, of whom nearly 200 million suffer from considerable functional difficulties [6]. In order to improve their living and working conditions, the WHO argues that it is necessary to promote empowerment and remove barriers that prevent them from participating in their communities, from having access to quality education, and taking up appropriate positions [6].

People with disabilities are now beginning to obtain recognition as a valuable resource [14,15,17]. Consequently, over the past two decades, there has been a significant increase in the inclusion of PWDs in the labor market, and the benefits for the individual, society, and organizations have been highlighted [20]. However, career opportunities for PWDs are still limited, because they suffer discrimination and prejudice at the hands of individuals, institutions, and society [21,22]. In addition, exclusion and negative attitudes can lead these people to illness, creating a substantial social burden [16,18].

Many of the problems that are faced by PWDs are not their own disabilities, but the physical and social barriers that keep them excluded [23], such as the negative attitudes and stereotyped views of colleagues and supervisors, workplace conflict, organizational culture, and climate [14].

As argued by Goffman [24], many of these problems occur because disability has historically been associated with stigmatization, prejudice, and discrimination. Prejudice is defined by Allport [25] as “an avertive or hostile attitude toward a person who belongs to a group, simply because he belongs to that group and is therefore presumed to have the objectionable qualities ascribed to the group” (p. 7).

According to Dovídio, Hewstone, Glick, and Esses [26], being prejudiced means having preconceived beliefs regarding groups of people or cultural practices. A person or group of people of specific political affiliation, gender, age, disability, and many other characteristics may suffer prejudice. These authors go on to argue that members of minority groups also develop prejudice towards members of the majority group, which constitutes a prior defense mechanism against the possibility of discrimination by members of the majority group.

Prejudice can negatively affect many aspects of the lives of PWDs. It can lead to self-depreciation, a distrust of the systems that evidence social stigma, difficulty in feeling included in the wider community, and self-isolation from others [27,28]. Prejudice can also work against opportunities for good jobs, safe housing, and adequate health care, and the ability to interact with different people and groups [29].

Prejudice and discrimination are among the main barriers faced by PWDs when entering the labor market, as pointed out by Neves-Silva, Prais, and Silveira [30] in relation to the Brazilian context. The International Labor Organization Convention (ILO) [31] considers discrimination as any distinction, exclusion, or preference that affects equality of opportunity or treatment in relation to employment or occupation. Discriminating against PWDs means treating them unfavorably because of their disability, when such attitudes cannot be objectively justified [32].

Discrimination affects PWDs in many ways. Globally, from the medical model of disability, mental illness is among the leading causes of disability and social exclusion [33]. Nevertheless, disability it is not only a medical status, but also a situation derived from the social construction of the impairment. In high-income countries, people with mental illness are frequently exposed to open and hidden actions of discrimination [34,35]. In Nigeria, the existence of widespread discrimination against PWDs in terms of job access and security has been identified [36]. In Spain, personal and group discrimination has been shown to harm self-esteem of PWDs in various ways [37].

The way in which disability is understood is important, because the that language people use to describe individuals with disabilities influences their expectations and interactions with them. The present research conceives disability as a social construct derived from the perceptions of society regarding the impairments of people with functional diversity [14].

### 2.2. Conceptions of Disability

Disability has been described, perceived, and treated in different ways in different historical periods [38,39,40,41]. At each stage of history, reference models emerged specifying how disability should be perceived.

According to Glat [42], disability was perceived for a long time as a form of chronic illness. Disabled individuals were considered to be incapable and invalid. This perception survived until the middle of the 20th century, when psychology research evidenced the need to recognize this group as a social construction.

Carvalho-Freitas [39,43] studied the literature on historical periods in order to construct the interpretative matrices of disabilities—Ancient Greece, Classical Period, Middle Ages, and Modern and Contemporary Periods—that directly or indirectly mentioned persons with disabilities. The author drew on several studies that refer to the issue of disability [38,40,41] in order to identify prevalent patterns of behavior towards PWDs in each historical period and what visions of human beings and the world might justify the origin of such conceptions. Carvalho-Freitas [39,43] identified six interpretative matrices of disability from this analysis.

Carvalho-Freitas [39] defines the conceptions of disability as interpretative matrices that, in a rational way or not, shape and validate categorizations and classifications of PWDs in the social and professional context. The author defines the interpretive matrices as “relatively stable and organized modes of thought anchored in conceptions of human beings, the world and society, which organize social activity, recognize and qualify needs, and admit ways of satisfying them, in function of their ends” (p. 35).

The first matrix is called subsistence/survival. In it, disability is perceived as an incapacitating obstacle to survival, because a “deformed body” implies that the individual is incapable of fulfilling his/her functions in society, thus becoming an obstacle to the functioning of the group. The principles that guide this matrix are implicitly present in all of the other matrices, which are based on the assumption that the ability of disabled individuals to attend to their basic needs determines their acceptance or not by the group.

The second matrix refers to the instrumental function of the person, which revolves around the search for an ideal organization and a “perfect and beautiful human being” who has a defined function in society. This matrix can be inserted into the historical context of Ancient Greece, between the 6th century BC and year 322 BC, when the philosophical thinking of Plato and Aristotle prevailed. In this period, which was marked by the weakening of mythical beliefs and the rise of city-states, a clear distinction was developed between the freemen dedicated to the worship of the body and leisure and the slaves who guaranteed the necessary infrastructure for social development.

The third matrix conceives disability as a spiritual phenomenon, of metaphysical origin, interpreting it as a manifestation of divine will or punishment. This matrix is observed in the Middle Ages. After the fall of the Roman Empire, the Judeo-Christian theories began to prevail in Western society. This context provides an understanding of the human body that is based on biblical references, conceiving disability as brought about by evil spirits, sins, witchcraft, or God’s displeasure.

The fourth matrix, entitled Normality, identifies disability as a deviation from the patterns that are regarded to be normal by society. It originated in the Modern Age, in the transition from feudalism to capitalism, with the rise of the scientific conception of the world and of man [44]. With the advance of medicine in this period, the spiritual conceptions of disability were replaced by its consideration as a manifestation of disease and, therefore, it became the physicians’ work to identify prognoses and methods of treatment. According to the ideas of John Locke (1632–1704), which affirm that experience is the foundation of all knowledge, mental deficiency is conceived as a stage that is marked by a lack of ideas and intellectual operations, similar to that of a newborn baby, which can be overcome by experience and teaching [40]. This matrix of interpretation maintained its hegemony for several decades. In Brazil, it was predominant from the 1960s to 1980s. However, nowadays, the perspective of social integration of PWDs through rehabilitation and their adaptation to the social system is still current [45]. Instead of valuing their potential for work when considering the insertion of PWDs in a company, this matrix uses disability itself as a criterion for placement and the allocation of responsibilities, as well as advocating for the segregation of PWDs in specific areas of the company that are separated from the other employees.

The fifth matrix is called Inclusion. It describes disability as a social rather than an individual problem. This matrix has its origins in the social movements of the twentieth century, especially after the English Industrial Revolution, characterized by the apogee of capital, the rise of the trade union movements, and the manifestations of minority groups struggling to guarantee civil rights for all. In this perspective, disability is conceived as a socio-political construction centered on a discourse based on social rights. Therefore, the approval of laws aimed at establishing a discourse based on rights rather than segregation [46] occurred in several Western countries.

Finally, the sixth matrix is referred to as the Technical Interpretation of Disability, according to which diversity is a resource to be managed and controlled by organizations. In this perspective, the approach to social differences shifts from political considerations to the management of human resources. Thus, diversity begins to be interpreted as a resource that, if well managed, can become a competitive advantage for both the company and individuals [46,47].

The way that these models interpret disability legitimizes the differentiation of PWDs in the groups where they are inserted, which offers a justification for the identified social positions [39,43].

Lastly, the understanding of the process of social inclusion is intrinsically linked to how individuals perceive the disability and the relationship between the disabled individual and society. This relationship is even more complex in the organizational context, while taking into account that it raises questions regarding how the organization should manage this group, providing it with adequate conditions for its professional development without, however, increasing situations of conflict in the work environment.

## 3. Materials and Methods

### 3.1. Participants

The respondents were managers of a Brazilian public organization and they were accessed by a non-probabilistic sampling method. The sample consisted of 257 respondents (participation rate: 14.5%), all of whom occupied managerial positions in operational (17%), support (28%), and customer service areas (55%). The majority were males (65.1%), married (74.6%), aged between 31 to 40 years old (38.3%), with academic degrees (43.8%), and having worked from 11 to 15 years in the organization (21.2%). Among the respondents, 3.9% had some kind of physical disability and 69.6% were working or had already worked with PWDs.

### 3.2. Instruments

Data were accessed by a survey-based method, applying a two-part questionnaire. The first part corresponded to the Disability Conceptions Inventory (DCI), created and validated in Portuguese by Carvalho-Freitas [39] in order to identify the conceptions of disability in organizations. It is composed of twenty-two items and seven factors (the English names of the factors are translated from the original version in Portuguese). The items have a six-response option on a Likert scale, which ranges from ‘totally disagree’ to ‘totally agree’. The DCI seven factors are described below:

Spiritual Conception of Disability (SCD): this consists of three items (e.g., Disability is a manifestation of a divine power that defines the characteristics that human beings should possess in their earthly life) with factor loadings that ranged from 0.50 to 0.86 and Cronbach’s Alpha = 0.64 [39]. The predominance of a high degree of agreement on this factor indicates a charitable attitude towards people with disabilities.

Conception Based on Assumptions of Normality (CAN): this consists of five items (e.g., only some sectors are suitable for people with disabilities to work in) with factor loadings ranging from 0.45 to 0.70 and Cronbach’s Alpha = 0.65 [39]. This factor focuses on people’s perceptions of the deviation from normality of PWDs and its implications for employment.

Conception Based on Assumption of Inclusion (CAI): this consists of two items (e.g., Persons with disabilities may adequately perform any type of work provided that the working conditions are adapted to their needs) with factor loadings of 0.50 and 0.81 and Cronbach’s Alpha = 0.53 [39]. This factor focuses on the perception of the need to adjust instruments and working conditions in order to facilitate the inclusion of PWDs in the company and the workplace.

Perception of Performance (PP): this consists of five items (e.g., people with disabilities perform at as well at work as non-disabled persons) with factor loads ranging from 0.57 to 0.76 and Cronbach’s Alpha = 0.77 [39]. This factor focuses on people’s perceptions of PWDs performance, productivity and quality of work, and their implications for company competitiveness.

Perception of Benefits of Hiring (PBH): this consists of three items (e.g., hiring people with disabilities improves the company’s image among the customers) with factor loadings ranging from 0.69 to 0.85 and Cronbach’s Alpha = 0.81 [39]. This factor focuses on the perception of the impact of hiring people with disabilities on the company’s image among employees and customers and on the organizational climate.

Perception of Bonding (PB): this consists of two items (e.g., people with disabilities are more committed than others) with factor loadings of 0.75 and 0.87 and Cronbach’s alpha = 0.79 [39]. This factor focuses on people’s perceptions of PWDs commitment and employment stability.

Perception of Training Needs (PTN): this consists of two items (e.g., leaders are not adequately trained to oversee the work of people with disabilities) with factor loadings of 0.77 and 0.82 and Cronbach’s Alpha = 0.62 [39]. This factor focuses on the perception of the need for training of managers and employees in order to integrate PWDs in the company and workplace.

The first three factors (SCD, CAN, CAI) are linked to the spiritual, normality, and inclusion matrices, and the other four factors (PP, PBH, PB, PTN) to the technical matrix.

The second part consisted of eight variables that were related to the demographic profile of the participants: gender; age; whether he/she was a PWDs; area where he/she was working; academic qualifications; how long he/she had worked for the company; marital status; and, whether the participant was working or had already worked with PWDs.

Firstly, we conducted a confirmatory factor analysis (CFA). The CFA results indicate a good fit for the seven-factor model (χ^2^ = 345.535, *p* < 0.001, df = 188; RMSEA = 0.057; SMRM = 0.0494; CFI = 0.931; IFI = 0.933).

We analyzed the convergent validity and reliability of the factors in order to evaluate the measurement validity of the instruments. The values of the average variance extracted (AVE) were all higher than 0.5 [48], except for Conception Based on Assumption of Normality. The construct reliability (composite reliability) and alpha coefficients were greater than 0.7 [49] (Table 1), except for Perception of Bonding, with alpha 0.62. Nevertheless, Loewenthal [50] suggested that this value is adequate for scales with less than 10 items.

Finally, discriminant validity was evaluated [51]. Table 2 shows the correlations between each pair of latent variables. The results show that these correlations are lower than the square root of AVE (on diagonal), indicating that the constructs measured by each scale are significantly different from the others [51].

### 3.3. Procedure

Firstly, a letter was sent to the company planning department, seeking authorization to conduct the research. Once permission was granted, all of the managers and their work areas were mapped. Subsequently, letters were sent to the managers of each area, informing them of the beginning of data collection and explaining the research objectives.

The questionnaire was distributed electronically, together with a letter indicating the research objectives, the researchers’ names, and their commitment to confidentiality and the anonymity. The respondents were guaranteed the confidentiality of individual responses and voluntary participation in the research, with free and informed consent.

### 3.4. Data Analysis

The data were first submitted to an exploratory analysis in order to identify the existence of missing values and outliers, as recommended by Hair, Anderson, Tatham, and Black [52]. The descriptive analysis was carried out by calculating the absolute and the relative frequencies of the sample. The mean, the standard deviation, and bootstrap 95% confidence interval were used in order to describe and compare the items and indicators of each construct. The Likert scale of concordance was set to vary from −1 (totally disagree) to 1 (totally agree). Spearman’s correlation was used to identify the association between the indicators.

To identify groups of managers according to their conceptions of disability, hierarchical cluster analysis and the Euclidean distance were used as a measure of dissimilarity [52]. All of the analyses were performed using the statistical package PASW Statistic, version 20.0.0.

## 4. Results

### 4.1. Managers’ Conceptions of Disability

After data analysis was completed, the indicators were described. as shown in Table 3, according to the Disability Conceptions Inventory (DCI) that was developed by Carvalho-Freitas [39]. The results show that the respondents disagree with the statements that formed the following indicators: Spiritual Conception of Disability, Conception Based on Assumption of Normality, and Perception of Performance. In contrast, they agree with the statements that formed the Indicators Conception Based on Assumption of Inclusion, Perception of Benefits of Hiring, Perception of Bonding, and Perception of Training Needs.

The analysis of the confidence intervals reveals that the indicator Perception of Training Needs presents a significantly higher mean (0.66) than the others, and the indicator Perception of Performance presents a significantly lower mean (−0.67) than the other indicators. This result shows that the majority of respondents have an inclusive view of the PWDs, since they agree with the Matrix of Social Inclusion. They perceive the inclusion of PWDs as a benefit to the organizational image and believe that PWDs are committed to the organization, not performing worse than other employees. They also consider it to be important to train the team to facilitate inclusion.

The Spearman correlation was used in order to verify the association between the managers’ conceptions of disabilities and their perception of the performance of PWDs, benefits of hiring PWDs, bonding, and training needs, as shown in Table 4. This correlation is a limited measure between −1 and 1, and the closer the coefficient is to −1, the greater the negative correlation, and the closer the coefficient is to 1, the greater the positive correlation. A correlation coefficient that is statistically equal to zero suggests the inexistence of a correlation between the two indicators tested.

The Spiritual Conception of the Disability showed a positive and significant correlation with the Perception of Benefits of Hiring and Perception of Bonding, according to Table 4. Thus, managers with a spiritual conception of disability perceive that hiring PWDs benefits both the organizational climate and the image.

The Conception Based on Assumptions of Normality showed a positive and significant correlation with the Perception of Performance. Thus, managers whose conception of disability is based on assumptions of normality perceive PWDs performance as inferior to that of other employees.

The Conception Based on Assumptions of Inclusion showed a positive and significant correlation with the Perception of Benefits of Hiring and Perception of Bonding, but a negative and significant correlation with Perception of Performance. Thus, managers who have this conception of disability tend to perceive the insertion of PWDs in the organization as beneficial to both the organizational climate and organizational image. These managers also believe that PWDs performance is not inferior to that of non-disabled employees.

### 4.2. Managers’ Conceptions of Disability and the Process of Inclusion

Table 5 presents the characterization of the groups that were formed by the cluster analysis. It should be noted that all of the indicators discriminate significantly (*p* < 0.05) in the groups, with the exception of the indicators of Spiritual Conception of the Disability and Perception of Training Needs, which presented *p*-values of 0.294 and 0.073, respectively. Two main groups emerged: Group 1—supporters of the inclusion of PWDs in the organizational space; and, Group 2—supporters of the insertion of PWDs in restricted sectors of the organization.

Group 1 is characterized by its presentation of the highest averages of the indicators based on assumptions of Inclusion, Perception of Benefits of Hiring, Perception of Bonding, and the lower averages of the indicators based on assumptions of Normality and Perception of Performance. These results reveal that the individuals in this group perceive disability as a social phenomenon where the environment must adjust to the needs of PWDs. so that they can fully participate in social actions. Disabled individuals should be included in the work environment according to their potentialities, and this group disagrees that their performance is inferior to that of other workers. In conclusion, this group has an inclusive view of PWDs.

**Figure 1:**
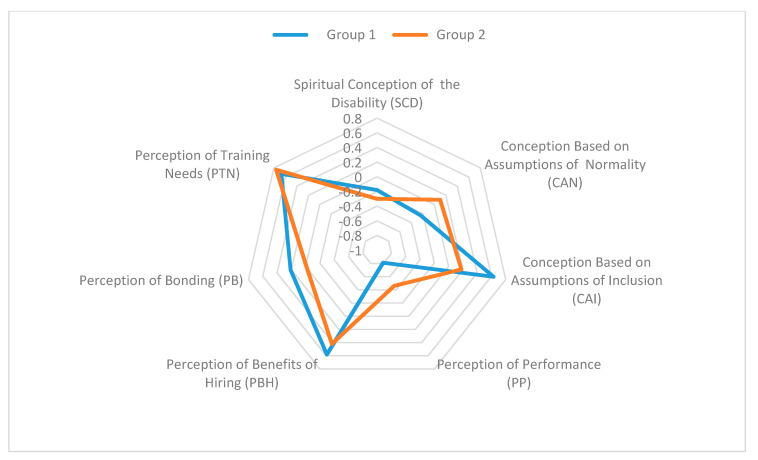
Star chart of profiles of managers.

Group 2 is characterized by its presentation of the highest averages for the indicators Conception based on the Assumption of Normality, and Perception of Performance, and the lowest averages for the indicators based on the assumptions of Inclusion, Perception of the Benefits of Hiring, and Perception of Bonding. These results reveal that the individuals in this group perceive disability as a “deviation” from normality. Disabled persons may perform inadequately in the workplace and may well increase the risk of accidents. Therefore, they must be allocated to specific sectors of the organization. PWDs performance is perceived to be lower than that of non-disabled employees. In conclusion, the individuals in this group have a segregationist view and negatively assess the potentialities of disabled persons. Figure 1 shows the profiles of the managers according to their conceptions of disability.

## 5. Discussion

The results show that the respondents tend to disagree with the conception of disability as a divine manifestation (moral model). However, they adopt a charitable attitude to PWDs. This, according to Carvalho-Freitas [39], could hamper workplace relations because of the treatment handed out to PWDs. These findings also reinforce the author’s premise that, although this conception has its origins in the Middle Ages, its manifestation can still be observed at several later points in history.

Even though the majority of the respondents disagree with the conception of disability in relation to assumptions of normality, part of the group still understands disability as an individual phenomenon that needs to be isolated and treated by specialized professionals, thus confirming the conclusions of Carvalho-Freitas [39]. According to this perspective, disability is the criterion that determines the allocation of PWDs to certain areas and tasks within the organization and not their potential.

The majority of respondents disagree that disability is a disease or a deviation from normality, and that the performance of PWDs is lower than non-disabled individuals (medical model). They tend to agree that hiring PWDs represents an advantage for the organization in terms of improving the organizational climate and the institutional image. However, they do not have a clear perception regarding the idea of segregation and inclusion of PWDs at work and in the company. These findings suggest that the work relationships of PWDs and non-disabled individuals are a complex phenomenon that demands a deeper understanding.

The managers do not consider that disability is a determining factor in itself when establishing a link between PWDs and hiring companies. Contextual factors must also be taken into account in this process. These results are also in line with the findings of Carvalho [53].

Although the respondents agree that it is important to train managers and staff to facilitate the insertion of disabled persons into the work environment, as pointed out by Moore, McDonald, and Bartlett [54] during the recruitment interview process and, whilst working on the job, previous research by Carvalho-Freitas [39] revealed that managers of various Brazilian organizations were not convinced about the effectiveness of training. Consequently, the lack of consensus around this issue suggests that more research is needed in order to clarify it.

These results indicate that managers who have a conception of disability that is based on assumptions of inclusion (social model) tend to perceive the insertion of PWDs in the organization as beneficial to both the organizational climate and organizational image. In addition, they believe that PWDs performance is not inferior to that of non-disabled employees.

These results have many implications. Managers who adopt a charitable attitude towards PWDs can make it difficult to establish good social relationships in their teams due to the different treatment given to PWDs and other workers. In these cases, the employee with disabilities is not perceived as a fully-fledged employee, but rather as a person to take care of. His/her inclusion in the team would be difficult and the risk of turnover would be high.

Given that managers do not have a homogeneous perception of the segregation and inclusion of PWDs at work and in the company, it may lead them to adopt disability as the primary criterion for the placement of PWDs rather than their potential.

The lack of consensus about the source of the disability may hinder inclusion. Managers who view disability as a social problem are likely to place workers with disabilities according to their potential. Those who have doubts about whether disability is an individual or social problem may have greater difficulties in placing PWDs in teams under their supervision. This issue prompts a need for senior managers to develop an internal guidance program in order to make the inclusion of PWDs at work and in the organization more effective.

The fact that most managers have a positive view of PWDs performance provides an opportunity to make the inclusion of these individuals in the company a natural process, as in the case of other employees.

Finally, the present research has some limitations that should be taken into account in future research. Firstly, the fact that the literature on this subject is not conclusive regarding the definition of disability and how it should be managed implies that further research is needed to clarify these matters [6,9,10,41]. Secondly, the factor in the instrument, called Conception Based on Assumption of Normality, had a low AVE level. Nevertheless, the rest of the adequacy indicators were satisfactory. Thirdly, this research was carried out in a public company in Brazil. A further study on an international scale is needed in order to test the validity of the results. Finally, the number of participants did not allow for analyzing the differential effect of age, gender, ethnicity, and other sociodemographic variables [55]. Future research should analyze the effect of these variables on individuals’ perceptions.

## 6. Conclusions

In its first stage, this research explored managers’ conceptions of disability in a large public organization. It then went on to identify the relations that were established between the conceptions of disability (spiritual, normality, and inclusion) and the matrix of technical interpretation of the deficiency (perception of performance, perception of bonding, perceptions of hiring benefits, and perception of training needs).

Three conceptions of disability were identified: the spiritual conception, the conception based on the assumption of normality, and the conception based on the assumption of inclusion, with predominance of the last one.

The managers disagree with the conception of disability as a divine manifestation, i.e., as the consequence of divine will or punishment. However, they do adopt a charitable attitude toward PWDs. This supports Carvalho-Freitas’s [39] idea that, even if this attitude originated in the Middle Age, it is still alive in people’s minds even today.

Although most managers disagree with the premise that disability is a disease or a deviation from normality, some of them perceive the existence of segregation in specific areas of the organization. There is no consensus regarding the causes of disability. Over fifty percent of the participants believe that disability is a social problem, while the others have doubts regarding whether disability is an individual or social problem.

Most managers disagree that PWDs performance is lower than that of non-disabled workers. They agree that many contextual factors interfere in the relationship between disabled people and the hiring organizations. Therefore, disability is not necessarily a determining factor in this relationship. Managers perceive a need to train managers and other employees in order to facilitate the inclusion of PWDs in the work environment.

Managers with a spiritual conception of disability and conception based on the idea of inclusion perceive the insertion of PWDs as beneficial to both the organizational climate and organizational image.

Managers who base their conception of disability on assumptions of inclusion do not perceive the work done by PWDs as inferior to that of other workers. Contrariwise, managers who base their conception of disability on the assumption of normality do perceive PWDs performance as inferior to that of other employees.

Two well-defined groups of managers were identified regarding attitudes to the insertion of PWDs into the work environment. One group considers that PWDs should be inserted into the work environment according to their potential. The other group has a segregationist view; they believe that disability is an individual problem and PWDs must therefore be segregated in the work environment and treated by specialized professionals.

The results of this research corroborate previous research [39,46,53,56] and, thereby, contribute to the understanding of how to manage the inclusion of persons with disabilities in the labor market.

## Figures and Tables

**Table 1:** Convergent validity and reliability.

Construct		Items	AVE ^1^	CA ^2^	CR ^3^
Disability Conceptions Inventory	Spiritual Conception of the Disability (SCD)	3	0.58	0.79	0.80
	Conception Based on Assumption of Normality (CAN)	5	0.33	0.71	0.71
	Conception Based on Assumption of Inclusion (CAI)	2	0.70	0.82	0.82
	Perception of Performance (PP)	5	0.65	0.90	0.90
	Perception of the Benefits of Contracting (PBC)	3	0.63	0.78	0.83
	Perception of Bond (PB)	2	0.58	0.62	0.71
	Perception of Training Needs (PTN)	2	0.70	0.82	0.82

^1^ Average Variance Extracted; ^2^ Cronbach’s alpha; ^3^ Composite Reliability.

**Table 2:** Discriminant validity.

	SCD	CAN	CAI	PP	PBC	PB	PTN
Spiritual Conception of the Disability (SCD)	0.76						
Conception Based on Assumption of Norm (CAN)	0.23 **	0.58					
Conception Based on Assumption of Inclusion (CAI)	0.03	−0.32 **	0.83				
Perception of Performance (PP)	0.11	0.39 **	−0.36 **	0.80			
Perception of the Benefits of Hiring (PBH)	0.19 **	−0.09	0.34 **	−0.22 **	0.80		
Perception of Bond (PB)	0.14 *	0.02	0.17 **	0.03	0.28 **	0.80	
Perception of Training Needs (PTN)	0.09	0.09	0.05	−0.02	0.17 **	0.05	0.76

Note: The square root of AVE is reflected on diagonal. * *p* ≤ 0.05; ** *p* ≤ 0.001.

**Table 3:** Description of the indicators.

Indicator		Mean	S.D. ^1^	C.I-95% ^2^
Disability Conceptions Inventory	Spiritual Conception of the Disability (SCD)	−0.27	0.57	[−0.34; −0.20]
	Conception Based on Assumption of Normality (CAN)	−0.16	0.42	[−0.21; −0.11]
	Conception Based on Assumption of Inclusion (CAI)	0.49	0.48	[0.44; 0.55]
	Perception of Performance (PP)	−0.67	0.38	[−0.72; −0.63]
	Perception of the Benefits of Hiring (PBH)	0.47	0.38	[0.43; 0.52]
	Perception of Bonding (PB)	0.07	0.42	[0.02; 0.11]
	Perception of Training Needs (PTN)	0.66	0.37	[0.61; 0.70]

^1^ Standard Deviation; ^2^ Bootstrap Confidence Interval.

**Table 4:** Correlation between the indicators created.

Indicator	Spiritual Conception of Disability (SCD)	Conception of Normality (CAN)	Conception of Inclusion (CAI)
Perception of Performance (PP)	0.12	0.39 *	−0.37 *
Perception of Benefits of Hiring a PWDs (PBC)	0.19 *	−0.08	0.33 *
Perception of Bonding (PB)	0.14 *	0.04	0.16 *
Perception of Training Needs (PTN)	0.08	0.08	0.06

* *p* < 0.05.

**Table 5:** Characterization of the groups formed.

Indicator	Group 1		Group 2		*p*
	Mean	E.P.	Mean	E.P.	
Spiritual Conception of the Disability (SCD)	−0.18	0.06	−0.30	0.07	0.294
Conception Based on Assumptions of Normality (CAN)	−0.24	0.03	0.05	0.06	0.000
Conception Based on Assumptions of Inclusion (CAI)	0.63	0.03	0.18	0.07	0.000
Perception of Performance (PP)	−0.81	0.02	−0.46	0.06	0.000
Perception of Benefits of Hiring (PBH)	0.58	0.03	0.42	0.05	0.008
Perception of Bonding (PB)	0.21	0.03	−0.01	0.05	0.000
Perception of Training Needs (PTN)	0.67	0.03	0.76	0.03	0.073

Source: Research Data.
